# BISON (Bloodstream Infections and Sepsis Outcomes measurement Network) initiative: an ESGBIS proposal

**DOI:** 10.1186/1745-6215-16-S1-P3

**Published:** 2015-05-29

**Authors:** Luigia Scudeller, Jesús Rodríguez Baño, Winfried V Kern

**Affiliations:** 1 Clinical Epidemiology and Biostatistics Unit, Scientific Direction, IRCCS Policlinico San Matteo, Pavia, Italy; 2 Infectious Diseases, Microbiology and Preventive Medicine, Hospital Universitario Virgen Macarena and Virgen del Rocío, Seville, Spain; 3 Division of Infectious Diseases, Albert-Ludwigs-University, Freiburg, Germany

## Motivation

Outcome of a bloodstream infection/sepsis (BSI/S) is defined in different ways in different research projects.[1] In this, BSI/S share the characteristics of other fields of medicine; specific issues are related to diversity of causative agents, the clinical focus of infection, severity of comorbid conditions, presence of indwelling devices, multiplicity of affected patient populations, and others.[2] A preliminary list of putative core outcome set (COS) might be:

• mortality

• time-to-clinical stability

• time-to-microbial clearance

• development/resolution of metastatic foci

• adverse events of treatment

• salvage of infected device

• relapse

• compliance

Therefore, the European Society of Clinical Microbiology and Infectious Diseases working group on bloodstream infections and sepsis (ESGBIS) is currently designing a project for the development of a COS in BSI/S.

## Objectives

1. generate a comprehensive long-list of all outcome variables reported in recent randomised controlled studies on BSI/S

2. refine the outcome long-list into a COS agreed by key stakeholders

3. define the best measurement methods of proposed COS

4. identify unresolved issues for further research

5. publish the proposed COS in the relevant scientific journals

6. build a COS database with standard fields easily shared across studies

7. monitor uptake of the selected core outcomes in future relevant publications

## Methods

Overall timetable: 2 year from first meeting.

**Figure 1:**
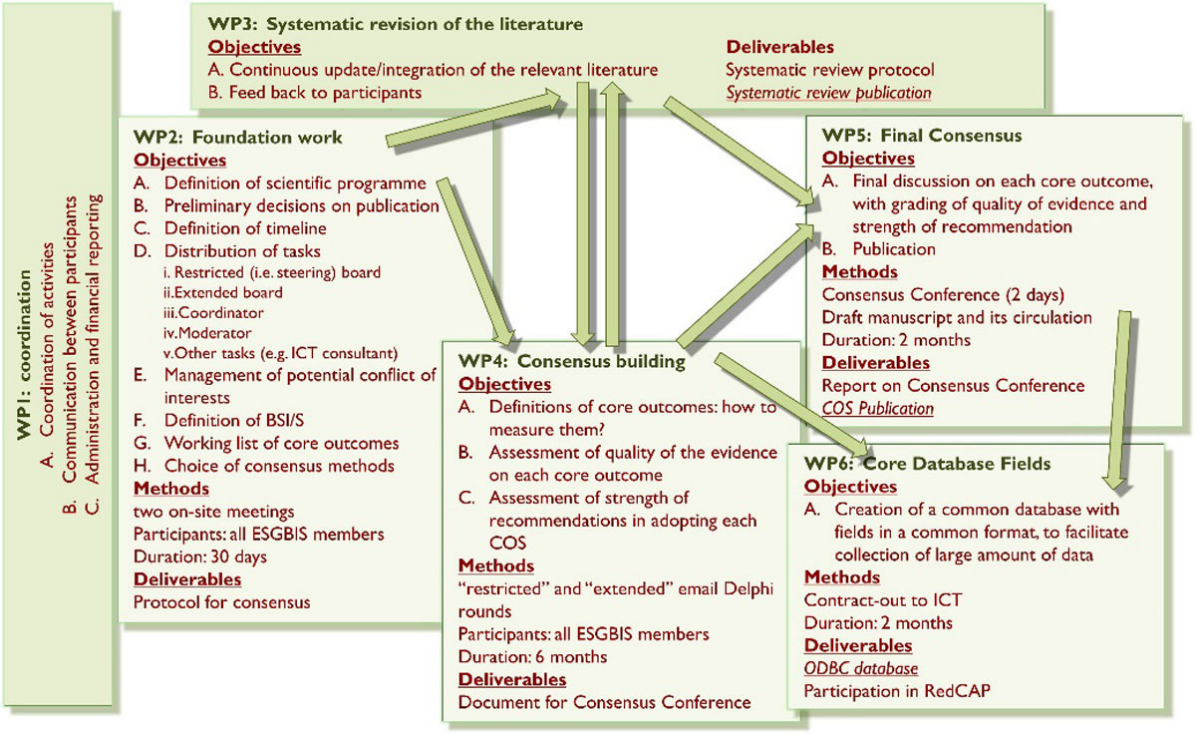
proposed structure of the BISON project

The ESGBIS group is composed by approximately 30 people with different backgrounds (infectious disease, microbiology, intensive care medicine, epidemiology and statistics). We will adopt a combination of methods: two preparatory meetings, restricted and extended Delphi rounds (via email exchanges), semi-structured interviews with patients, systematic review of the literature, final consensus conference (Figure 1).

## Expected outputs

Publication of the systematic review and of the COS in medical journals and development of a standard set of database fields, to be included in eCRF of future studies.
